# Exploring the role of breastfeeding, antibiotics, and indoor environments in preschool children atopic dermatitis through machine learning and hygiene hypothesis

**DOI:** 10.1038/s41598-025-94255-z

**Published:** 2025-03-21

**Authors:** Jinyang Wang, Haonan Shi, Xiaowei Wang, Enhong Dong, Jian Yao, Yonghan Li, Ye Yang, Tingting Wang

**Affiliations:** 1 Department of Clinical Medicine, Xinjiang Medical University, Urumqi, 830017 China; 2 The Zhoupu Affiliated Hospital of Shanghai University of Medicine and Health Sciences, Shanghai, 201318 China; 3 School of Nursing and Health Management, Shanghai University of Medicine and Health Sciences, No. 1500, Zhouyuan Road, Zhoupu Town, Pudong New District, Shanghai, 201318 China; 4 School of Public Health, Xinjiang Medical University, Urumqi, 830017 China; 5 Department of Geriatrics and Cadre Ward, The Second Affiliated Hospital of Xinjiang Medical University, No. 38, North 2nd Lane, Nanhu East Road, Shuimogou District, Urumqi, 830063 China

**Keywords:** Atopic dermatitis, Indoor environmental factors, Antibiotic use, Exclusive breastfeeding, Hygiene hypothesis, Machine learning, Skin diseases, Health care, Medical research, Risk factors

## Abstract

The increasing global incidence of atopic dermatitis (AD) in children, especially in Western industrialized nations, has attracted considerable attention. The hygiene hypothesis, which posits that early pathogen exposure is crucial for immune system development, is central to understanding this trend. Furthermore, advanced machine learning algorithms have provided fresh insights into the interactions among various risk factors. This study investigates the relationship between early childhood antibiotic use, the duration of exclusive breastfeeding, indoor environmental factors, and child AD. By integrating machine learning techniques with the hygiene hypothesis, we aim to assess and interpret the significance of these risk factors. In this community-based case–control study with a 1:4 matching design, we evaluated the prevalence of AD in preschool-aged children. Data were collected via questionnaires completed by the parents of 771 children diagnosed with AD, matched with controls based on gender, age, and ethnicity. Univariate analyses identified relevant characteristics, which were further examined using multivariable logistic regression to calculate odds ratios (ORs). Stratified analyses assessed confounders and interactions, while the significance of variables was determined using a machine learning model. Renovating the dwelling during the mother’s pregnancy (OR = 1.50; 95% CI 1.15–1.96) was identified as a risk factor for childhood AD. Additionally, antibiotic use three or more times during the child’s first year (OR = 1.92; 95% CI 1.29–2.85) increased the risk of AD, independent of the parents’ history of atopic disease and the child’s mode of birth. Moreover, exclusive breastfeeding for four months or more (OR = 1.59; 95% CI 1.17–2.17) was identified as a risk factor for AD, particularly in the group without a maternal history of atopic disease. In contrast, having older siblings in the family (OR = 0.76; 95% CI 0.63–0.92) and low birth weight (OR = 0.62; 95% CI 0.47–0.81) were identified as protective factors against AD. Machine learning modeling indicated that the duration of exclusive breastfeeding, having older siblings, low birth weight, and parental history of AD or allergic rhinitis are key predictors of childhood AD. Our findings support the broader interpretation of the hygiene hypothesis. Machine learning analysis highlights the key role of the hygiene hypothesis and underscores the need for future AD prevention and healthcare initiatives focusing on children with a parental history of AD or allergic rhinitis. Moreover, minimizing antibiotic overuse may be essential for preventing AD in children. Further research is necessary to elucidate the impact and mechanisms of exclusive breastfeeding on AD to instruct maternal and child healthcare practices.

## Introduction

Atopic Dermatitis (AD) is a common chronic, recurrent skin disease characterized by dry skin, localized red scaly patches, intense itching, and skin pain^1,2^. AD affects patients’ quality of sleep, school and work, and even future career plans^3,4^, leading to reduced quality of life and increased healthcare expenditures^5^. For example, individuals with adult eczema who require out-of-pocket payments of 371–489 dollars per person year^6^ and those with hand eczema frequently face significant challenges while trying to unlock their fingerprints^7^. According to the most recent Global Burden of Disease (GBD) 2022 report, there are an estimated 223 million AD cases globally, with nearly 20% of cases occurring in children between the ages of 1–4. This represents a significant healthcare burden on society. Additionally, AD ranks first among skin diseases and 15th among nonfatal diseases in the Global Burden of Disease based on Disability-Adjusted Life Years (DALYs)^1,2,8^. AD has a complex pathogenesis involving gene-environment interactions, skin barrier disruption, microbial homeostasis disruptions, and immunoregulation imbalances^1,9,10^.

A significant amount of recent research has focused on identifying the genetic and environmental factors contributing to AD, such as parental asthma or AD^11^, FLG gene mutations^12^, the degree of hygiene^13^, air pollution^14^, ultraviolet light exposure^15^, diversity of intestinal flora^16^, immunization^17^, green cover, tobacco exposure^18^, and environmental hormonal persistent organic pollutants^19^. These recent findings have significantly enhanced our understanding of AD and contributed to the development of preventive strategies. However, considering the increased prevalence and incidence of AD over the past few decades^2,19^, it is important to assess if any crucial issues have been overlooked. Epidemiologists have discovered since the turn of the century that having many siblings^20^, attending daycare centers^21,22^, residing on farms with animals^23^, and having furry pets^24^ all offer varied degrees of protection against the onset of atopic illnesses. Possible explanations include early childhood close contact with other children or living in a rural area, which can increase exposure to a variety of microorganisms and pathogens, thereby partially triggering the natural maturation process of the immune system. On the other hand, AD may arise from an imbalance between type 1 helper cells (Th1) and type 2 helper cells (Th2)^25^. High hygienic cleanliness scores were associated with an increased risk of reporting AD at 30–42 months of age (OR = 1.04, 1.01–1.07), particularly for AD with painful, exudative fluid, as indicated by studies on the relationship between hygienic cleanliness in infancy and AD^13^. Therefore, while improved cleanliness has notably reduced the incidence and mortality of infectious diseases, it may also contribute to the ongoing rise in the prevalence of AD, a concept known as the “hygiene hypothesis” proposed by Strachan^26^.

Machine learning, a branch of artificial intelligence, relies on advanced statistical algorithms to identify inherent patterns in extensive datasets^27^. This capability allows researchers to uncover novel insights, including the importance of specific variables and the interrelationships among various risk factors—insights often beyond the reach of traditional statistical methods such as logistic regression^28^. Machine learning models employing various algorithms explore the effect of the variable of interest on the outcome from multiple perspectives, providing a more comprehensive understanding of the issue. Moreover, the robust predictive capacity of machine learning aids in identifying high-risk populations and delivering personalized preventive and treatment protocols, which are essential for the early prevention and diagnosis of atopic dermatitis. This research utilized a case–control study design to investigate the overall impact of various potential variables on early childhood AD and their relationship to the hygiene hypothesis. Additionally, a machine learning model was developed to evaluate the relative significance of these variables across different algorithmic frameworks.

## Materials and methods

### Study population

**Fig. 1:**
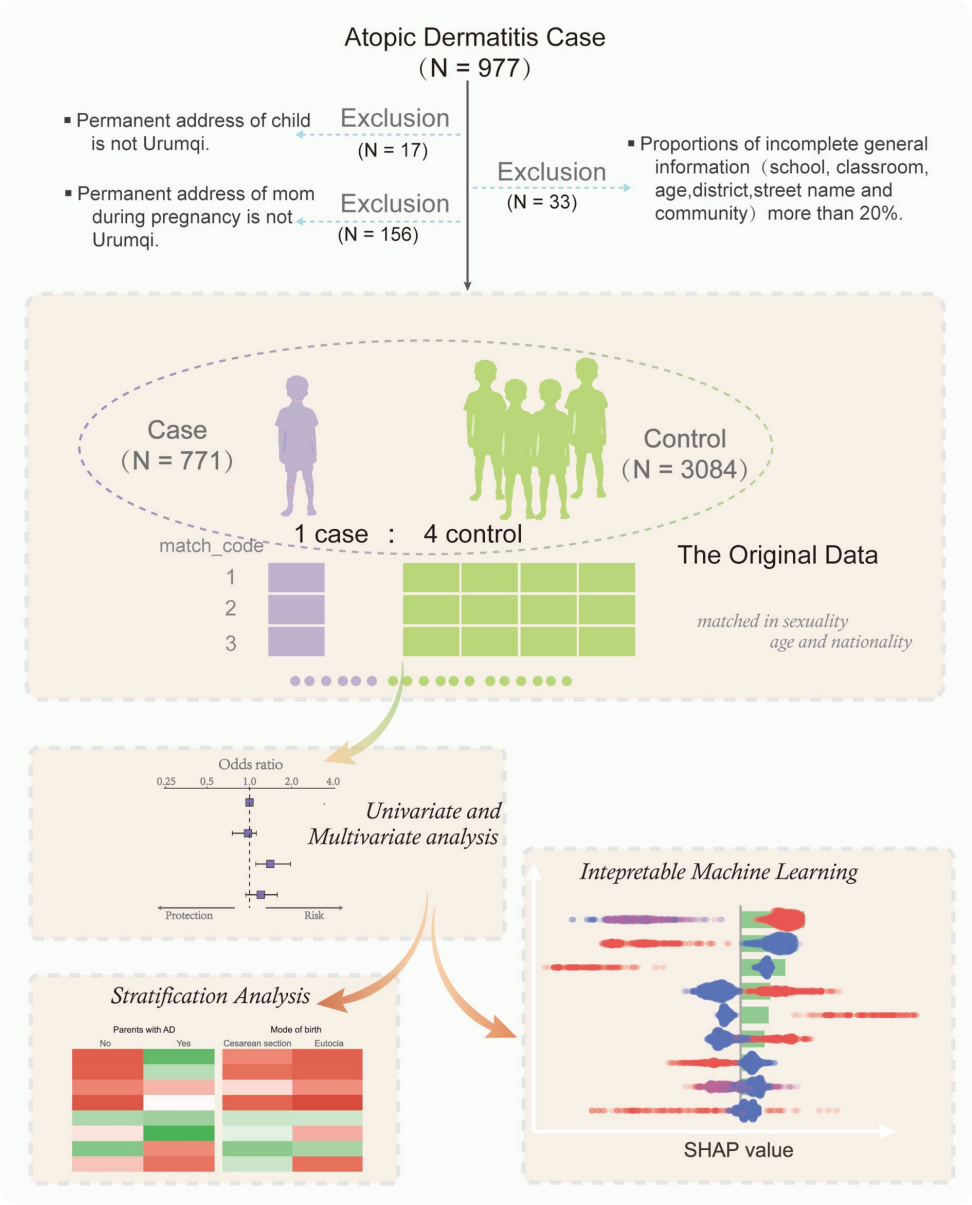
The flowchart of this study.

In August 2019, six administrative districts—Xinshi, Shayibake, Tianshan, Shuimogou, Toutunhe, and Midong—were selected from 40 districts in Urumqi using stratified random sampling. Subsequently, eight to twelve kindergartens, totaling sixty, were randomly chosen from each district to study children diagnosed with atopic dermatitis by a physician. Concurrently, four children without a diagnosis of AD were selected as controls and matched by gender, age, and ethnicity. Each child in the control group had an equal chance of participating in this investigation. The study was approved by the hospital’s ethical committee, and parents provided signed informed consent. A standardized questionnaire was developed and administered to all children in both the case and control groups. This questionnaire was adapted from the one used in the China Children, Homes, Health (CCHH) study, with minor modifications for Urumqi. The questionnaire using in this study comprised four sections: general demographic information, child feeding status, AD prevalence in children and family members, and living environment. Prior to the survey, the research team communicated with the Urumqi City Department of Education and kindergarten teachers, who then received standardized professional training. Preschool teachers agreed to distribute the questionnaire to the parents of the children, who were instructed to complete it at home. Parents were given one week to complete the questionnaire and submit it to the designated kindergarten teacher, who subsequently returned it to the Urumqi Education Bureau. All questionnaires were reviewed by at least two trained survey team members, and those deemed unqualified were excluded. The inclusion–exclusion process is illustrated in Fig. 1.

Key variables in this study were defined as follows: atopic dermatitis was determined by the question, “Has the child ever had a physician-diagnosed case of AD or eczema?”; antibiotic use was defined based on whether the child had received antibiotic treatment and the frequency of use during the first year. Breastfeeding status was assessed through questions regarding whether the child was exclusively breastfed by the mother and the duration of breastfeeding. Sibling status was evaluated by determining if the child was an only child; if not, the number of older and younger siblings was recorded. Additionally, we recorded each child’s birth weight, categorizing weights below 2.5 kg as low birth weight and those above 4.0 kg as fetal macrosomia.

### Statistical analysis

Epi Data 3.1 was used to establish the database, and R 4.3.0 software was used to analyze and process the data; the data were screened firstly by the inclusion and exclusion criteria, and non-random missing data were filled in according to the cause, random missing data were filled in by Multiple Imputation by Chained Equations (MICE)^29^. Univariate analyses were performed using the χ^2^ test. Factors associated with AD occurrence of preschool children were analyzed using multivariate logistic regression, with odds ratios (ORs) and 95% confidence intervals (CIs) reported. All statistical analyses were performed using the two-sided test criterion of α = 0.05, and P < 0.05 was considered statistically significant.

### Stratification analysis

To further explore whether the effects of variables of interest on outcomes in multifactorial analyses were influenced by intrinsic attributes, we conducted independent stratified analyses of intrinsic attribute variables such as parental history of allergy-related disorders including atopic dermatitis, allergic rhinitis, and asthma, parental history of allergic rhinitis or asthma, parental AD status, and child’s mode of birth. Considering the 1:4 matching of the data in this study, we used a unique strategy that distinguishes us from traditional stratified analyses, where we introduced the concept of Basic Data Unit (BDU), which is defined as a sample of 1 case and its 4 samples of healthy controls matched according to gender, age, and ethnicity. Our stratification strategy was to retain the BDUs that could be matched to the stratification variables and discard the BDUs that could not be matched, resulting in 1:1 case–control data based on the stratification variables, which included gender, age, ethnicity, and the stratification variables. Figure S1 shows an example of a Parent with atopic disease history, which can help to understand the stratified analysis strategy of this study.

### Interpretable machine learning models

Traditional statistical methods are limited when analyzing large, multidimensional datasets. Logistic regression is a variation of generalized linear regression that utilizes the sigmoid function for dichotomous classification, mapping input values to the [0,1] interval. While Logistic regression is straightforward, historically established, and effective for analyzing simple datasets, it often fails to fully capture underlying patterns in large datasets through coefficients. In contrast, Random Forest and eXtreme Gradient Boosting (XGBoost) are advanced ensemble models based on decision trees, differing primarily in their integration strategies for the base learners. Random Forest combines classification and regression trees (CART) with a parallel bagging strategy that enhances learner diversity through self-sampling and feature subset perturbation, culminating in a final decision based on voting. Its parallel architecture enables the use of the Gini index—a metric for evaluating node partitioning in CART—evolving into Mean Decrease Gini (MDG) in Random Forest. A higher MDG indicates greater importance of the feature for model performance. Conversely, XGBoost is a sequentially integrated model that adjusts data distribution based on preceding training results before training each base learner. It uses the outputs of weighted learners for final decision-making. Its sequential training process complements the SHAP interpretability framework. The SHAP value, grounded in game theory^30^, was initially applied to economic modeling before being successfully adopted in machine learning. In machine learning, SHAP assesses the significance of individual features by comparing model performance with and without them. This technique evaluates not only a feature’s relative importance but also its directional impact. The MDG from random forests can also assess feature importance; however, this metric primarily reveals quantitative significance.

The operational process encompasses various challenges, including feature engineering, handling class imbalance, optimal parameter tuning, and model iteration. Among these, class imbalance is a significant issue that impacts model performance. With a 1:4 case–control ratio in this study, the target variable accounts for only 20%. Complex machine learning algorithms typically require this ratio to be around 1:2. Given the unique advantages of BDUs in this study, we opted for down-sampling instead of up-sampling to address class imbalance. We implemented a computational strategy for sample spacing to retain control samples that differ significantly from case samples in each BDU. This approach maximizes the separation between case and control samples in feature space while maintaining the original design pattern of 1:1 matching in case–control studies. Figure S2 illustrates the data down-sampling strategy, aiding in the comprehension of the study’s machine learning component. Ultimately, we employed the area under the receiver operating characteristic curve (AUROC) as the primary metric for model performance evaluation, presented as mean ± standard deviation. All analyses were conducted using R (Version 4.3.0), with the following packages: mice (Version 3.16.0), caret (Version 6.0–94), randomForest (Version 4.7–1.1), xgboost (Version 1.7.6.1), and ggplot2 (Version 3.4.4). The Python Streamlit module (Version 1.32.0) was used for model deployment.

## Result

### Characteristics of the study population

The case group consisted of 771 children: 400 boys (51.9%), 371 girls (48.1%), 717 Han Chinese (93.0%), and 54 individuals from ethnic minorities (7.0%). The mean age of participants was 5.40 ± 1.06 years, with an average age of first atopic dermatitis episode at 2.16 ± 1.32 years. The control group comprised 3084 children matched in a 1:4 ratio based on similar gender, ethnicity, and age distribution to the case group. Significant differences emerged between the control and AD groups regarding mode of birth, full-term status, sibling presence, birth weight, paternal asthma, paternal allergic rhinitis (AR), paternal AD, maternal asthma, maternal AR, and maternal AD (P < 0.05). Detailed results are presented in Table 1.

**Table 1:** Demographic characteristics of ad and control groups.

Factors	Control group (%)	AD group (%)	χ^2^	P value
Mode of birth			4.75	0.0294
Eutocia	1475 (47.83%)	335 (43.45%)		
Cesarean section	1609 (52.17%)	436 (56.55%)		
Whether child was born at full term or not			6.10	0.0473
Yes	2341 (75.91%)	553 (71.73%)		
No, premature delivery	400 (12.97%)	122 (15.82%)		
No, post-term birth	343 (11.12%)	96 (12.45%)		
Siblings			11.86	0.0027
The only child	1765 (57.23%)	484 (62.78%)		
Have older siblings	747 (24.22%)	143 (18.55%)		
Have younger siblings	572 (18.55%)	144 (18.68%)		
Birth weight			16.93	< 0.001
Normal	2466 (79.96%)	651 (84.44%)		
Low birth weight (less than 2.5 kg)	396 (12.84%)	58 (7.52%)		
High birth weight (more than 4.0 kg)	222 (7.2%)	62 (8.04%)		
Father with asthma			11.26	< 0.001
No	3068 (99.48%)	758 (98.31%)		
Yes	16 (0.52%)	13 (1.69%)		
Father with AR			44.34	< 0.001
No	2274 (73.74%)	475 (61.61%)		
Yes	810 (26.26%)	296 (38.39%)		
Father with AD			60.41	< 0.001
No	2971 (96.34%)	690 (89.49%)		
Yes	113 (3.66%)	81 (10.51%)		
Mother with asthma			4.56	0.0327
No	3050 (98.9%)	755 (97.92%)		
Yes	34 (1.1%)	16 (2.08%)		
Mother with AR			69.44	< 0.001
No	2268 (73.54%)	449 (58.24%)		
Yes	816 (26.46%)	322 (41.76%)		
Mother with AD			106.61	< 0.001
No	2912 (94.42%)	642 (83.27%)		
Yes	172 (5.58%)	129 (16.73%)		

### Univariate analysis of indoor environmental factors, breastfeeding, and antibiotic exposure on AD

Table 2 displays the population distribution, χ^2^ values, and associated p-values for the AD and control groups. It was observed that multiple factors were significantly different between the two groups, including newly purchased furniture, renovations, mold presence, dampness in the parents’ residence prior to or during maternal pregnancy, and smoking by the father and maternal grandfather during the mother’s pregnancy. Additionally, factors such as newly purchased furniture and renovations during the child’s first year, mold presence, dampness, father and maternal grandfather smoking during this period, pet ownership, exposure to fish or reptiles, the duration of exclusive breastfeeding and antibiotics exposure were significantly different between the AD and control groups (P < 0.05). No significant differences were observed between the two groups regarding paternal grandfather smoking during maternal pregnancy, paternal grandfather smoking, ownership of dogs, or cultivation of flowering plants during the child’s first year (P > 0.05). The Cochran-Armitage trend test indicated a significant trend regarding the duration of exclusive breastfeeding and frequency of antibiotic treatment during the child’s first year across subgroups (P for trend < 0.01).

**Table 2:** One-way analysis of breastfeeding and antibiotic exposure in early life indoor environmental factors.

Factors	Control group (%)	AD group (%)	χ^2^	P value
Furniture was newly purchased in parents’ residence before mp			6.05	0.0139
No	2393 (77.59%)	566 (73.41%)		
Yes	691 (22.41%)	205 (26.59%)		
Renovation in parents’ residence before mp			4.8	0.0285
No	2619 (84.92%)	630 (81.71%)		
Yes	465 (15.08%)	141 (18.29%)		
Mold was noted in the parents’ residence before mp			13.32	< 0.001
No	2840 (92.09%)	678 (87.94%)		
Yes	244 (7.91%)	93 (12.06%)		
Dampness was noted in the parents’ residence before mp			10.1	0.0015
No	2974 (96.43%)	724 (93.9%)		
Yes	110 (3.57%)	47 (6.1%)		
Furniture was newly purchased in parents’ residence during mp			13.75	< 0.001
No	2799 (90.76%)	665 (86.25%)		
Yes	285 (9.24%)	106 (13.75%)		
Renovation in parents’ residence during mp			22.24	< 0.001
No	2927 (94.91%)	697 (90.4%)		
Yes	157 (5.09%)	74 (9.6%)		
Mold was noted in the parents’ residence during mp			17.12	< 0.001
No	2898 (93.97%)	692 (89.75%)		
Yes	186 (6.03%)	79 (10.25%)		
Dampness was noted in the parents’ residence during mp			5.17	0.023
No	2994 (97.08%)	736 (95.46%)		
Yes	90 (2.92%)	35 (4.54%)		
Father smoking during mp			9.82	0.0017
No	2404 (77.95%)	560 (72.63%)		
Yes	680 (22.05%)	211 (27.37%)		
Paternal grandfather smoking during mp			0.89	0.3465
No	2915 (94.52%)	722 (93.64%)		
Yes	169 (5.48%)	49 (6.36%)		
Maternal grandfather during mp			11.67	< 0.001
No	3027 (98.15%)	741 (96.11%)		
Yes	57 (1.85%)	30 (3.89%)		
Furniture was newly purchased in parents’ residence during cfy			7.69	0.0056
No	2847 (92.32%)	688 (89.23%)		
Yes	237 (7.68%)	83 (10.77%)		
Renovation in parents’ residence during cfy			10.26	0.0014
No	2953 (95.75%)	717 (93%)		
Yes	131 (4.25%)	54 (7%)		
Mold was noted in the parents’ residence during cfy			4.25	0.0393
No	2916 (94.55%)	714 (92.61%)		
Yes	168 (5.45%)	57 (7.39%)		
Dampness was noted in the parents’ residence during cfy			12.6	< 0.001
No	3007 (97.5%)	733 (95.07%)		
Yes	77 (2.5%)	38 (4.93%)		
Father smoking during the cfy			4.53	0.0334
No	2281 (73.96%)	541 (70.17%)		
Yes	803 (26.04%)	230 (29.83%)		
Paternal grandfather smoking during the cfy			0.5	0.4811
No	2836 (91.96%)	703 (91.18%)		
Yes	248 (8.04%)	68 (8.82%)		
Maternal grandfather smoking during the cfy			10.54	0.0012
No	3002 (97.34%)	733 (95.07%)		
Yes	82 (2.66%)	38 (4.93%)		
Keeping pets or growing plants at the child’s residence during cfy			10.81	0.001
No	2012 (65.24%)	454 (58.88%)		
Yes	1072 (34.76%)	317 (41.12%)		
Dogs was kept during cfy			1.23	0.2673
No	3034 (98.38%)	754 (97.8%)		
Yes	50 (1.62%)	17 (2.2%)		
Fishes or reptile was kept during cfy			18.78	< 0.001
No	2909 (94.33%)	694 (90.01%)		
Yes	175 (5.67%)	77 (9.99%)		
Flowering plants were grown during cfy			2.29	0.13
No	2283 (74.03%)	550 (71.34%)		
Yes	801 (25.97%)	221 (28.66%)		
Time of exclusive breastfeeding			10.76	0.0046
No	280 (9.08%)	44 (5.71%)	− 3.19*	0.0014*
1–3 months	528 (17.12%)	122 (15.82%)		
≥ 4 months	2276 (73.8%)	605 (78.47%)		
Times of antibiotic therapy during cfy			21.5	< 0.001
No	2158 (69.97%)	490 (63.55%)	− 4.13*	< 0.001*
1–2 times	881 (28.57%)	254 (32.94%)		
≥ 3 times	45 (1.46%)	27 (3.5%)		

*Cochran-Armitage trend test. *mp* maternal pregnancy, *cfy* child first year.

### Multivariate analysis of indoor environmental factors, breastfeeding, and antibiotic exposure on AD

**Fig. 2:**
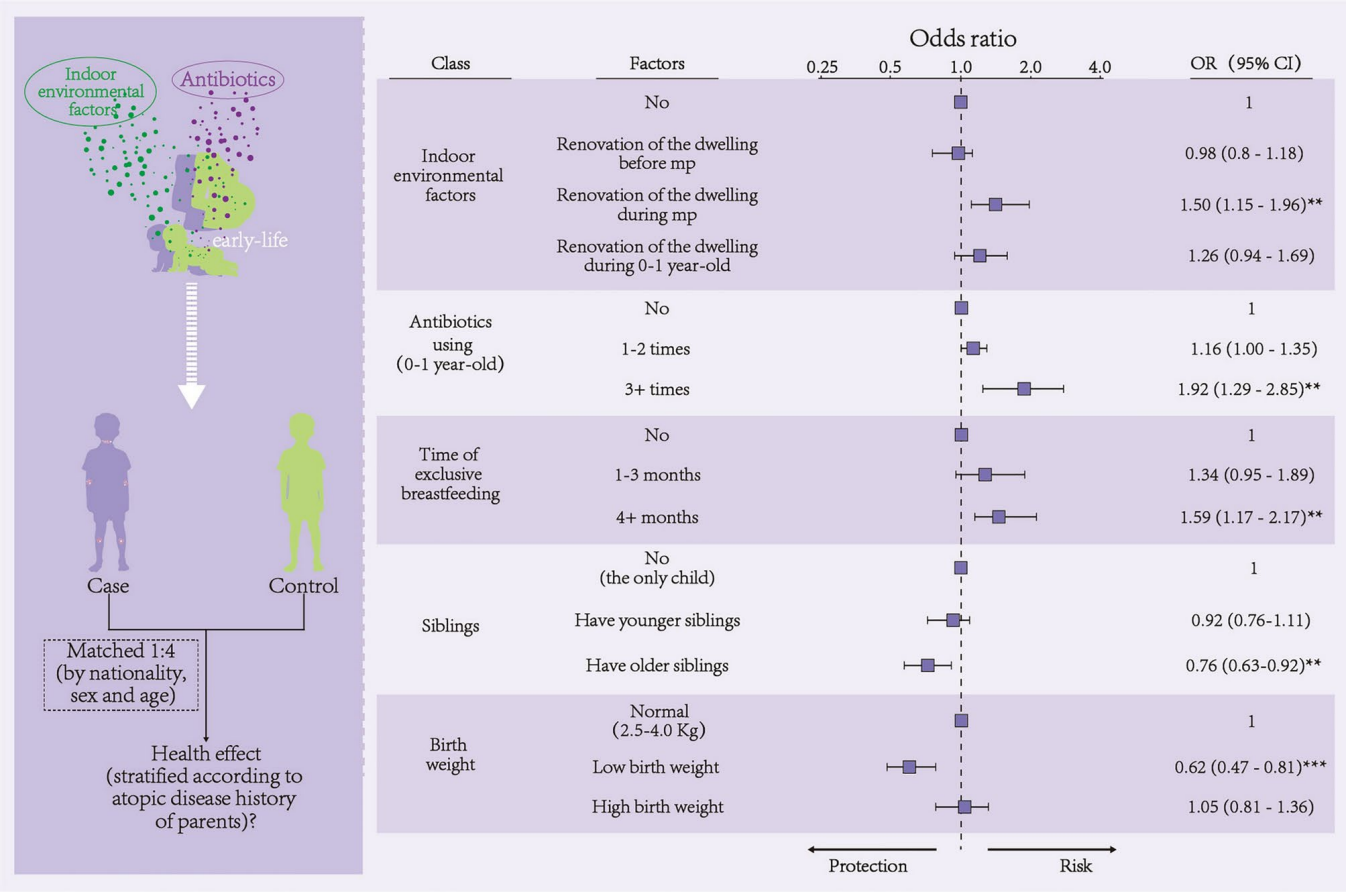
Multifactorial logistic analysis of indoor environmental factors, antibiotic use during the first year, breastfeeding, and sibling effects on preschoolers with AD; see Table S1 for complete results and covariates. *mp* maternal pregnancy, *cfy* child first year.

Variables demonstrating statistical significance (P < 0.05) in the univariate analysis were included in the logistic regression analysis. Table S1 lists the statistically significant variables included in the multifactorial analysis, along with their odds ratios (ORs) and 95% confidence intervals. The analysis revealed that paternal asthma (OR = 2.07, 95% CI 1.19–3.6), paternal AR (OR = 1.42, 95% CI 1.22–1.64), paternal AD (OR = 1.79, 95% CI 1.41–2.27), maternal AR (OR = 1.52, 95% CI 1.31–1.76), and maternal AD (OR = 2.10, 95% CI 1.72–2.55) are genetically associated risk factors for childhood AD. Figure 2, derived from Table S1, illustrates the results of the multivariate analysis concerning indoor environmental factors, antibiotic use, exclusive breastfeeding duration, sibling presence, and birth weight in relation to AD. Notably, renovation of the dwelling during maternal pregnancy was identified as an indoor environmental risk factor for AD (OR = 1.50, 95% CI 1.15–1.96). Our findings indicated that children receiving three or more antibiotic treatments between 0–1 year old had a significantly increased risk of AD compared to those who did not (OR = 1.92, 95% CI 1.29–2.85). Additionally, children exclusively breastfed for four months or longer also faced an elevated risk compared to those not exclusively breastfed (OR = 1.59, 95% CI 1.17–2.17). Furthermore, children with older siblings demonstrated a reduced risk of AD compared to only children (OR = 0.76, 95% CI 0.63–0.92). Moreover, children with low birth weight exhibited a lower risk of AD compared to those of normal weight (OR = 0.62, 95% CI 0.47–0.81).

### Stratified analyses by parental history of atopic diseases and child’s mode of birth

**Fig. 3:**
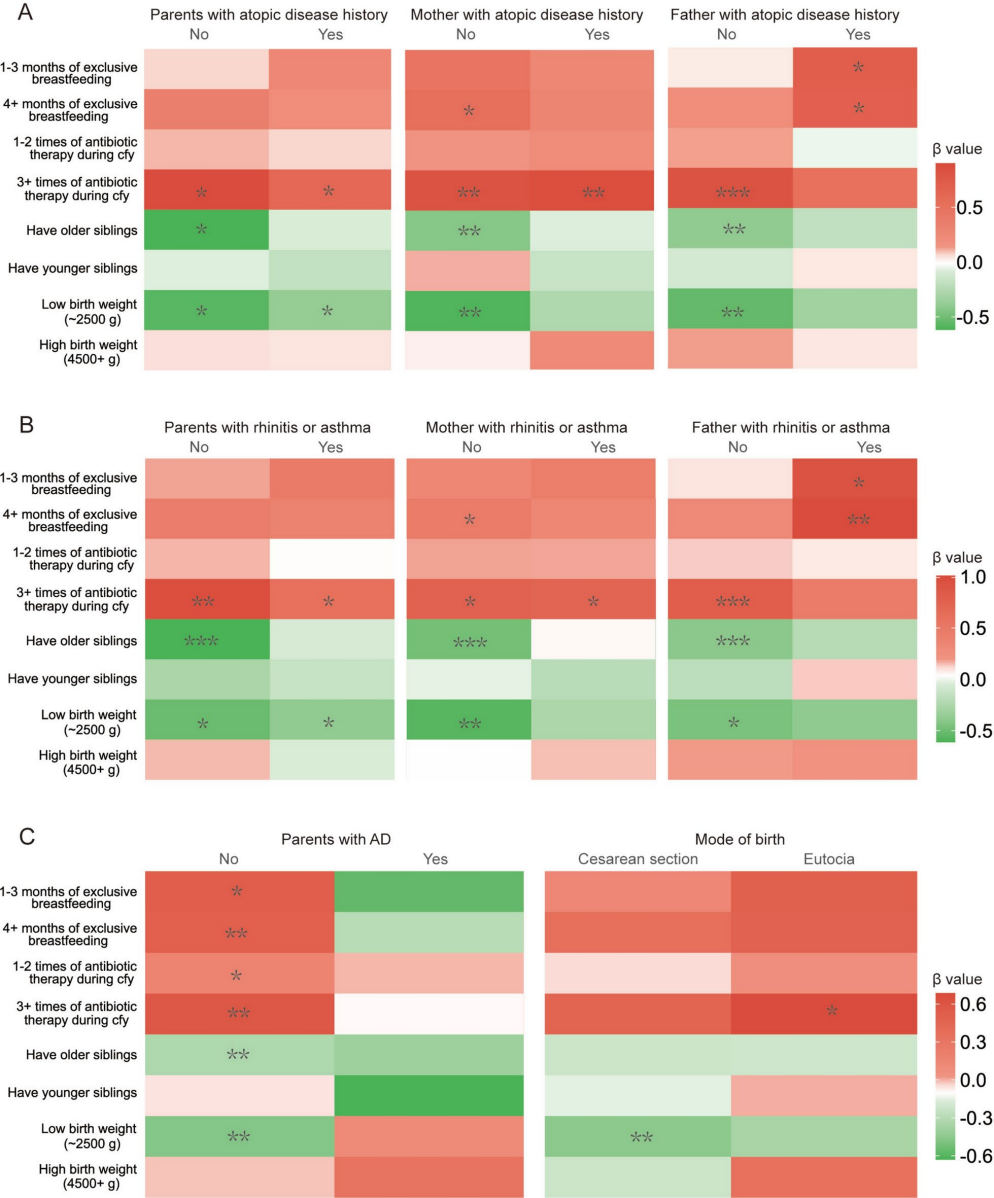
Stratified analysis of atopic disease history (**A**), rhinitis or asthma history (**B**), parental AD, and mode of birth (**C**) to explore factors influencing childhood AD, including exclusive breastfeeding duration, antibiotic use during the first year, sibling status, and birth weight.

For variables of interest during the multifactorial analysis, including exclusive breastfeeding duration, antibiotic using at age 0–1 years, sibling status, and birth weight, we conducted stratified analyses to analyze in depth whether the health effects of these variables on children’s development of AD were moderated by intrinsic attributes. Figure 3 shows the change in the effect of the variable of interest on the outcome after stratifying alone for parental history of atopic disease (Fig. 3A, Table S2), parental allergic rhinitis or asthma (Fig. 3B, Table S3), parental AD, and child’s mode of birth (Fig. 3C, Table S4), with covariates including all variables except stratification and the interest variable in Table S1. Overall, exclusive breastfeeding at 4 months and older and antibiotic use at 3 and older early in life showed a predominantly hazardous effect on AD, whereas having an older sibling and low birth weight showed a predominantly protective effect on AD. Specifically, we observed that the risk effect of exclusive breastfeeding for 4 months or more on AD was significantly lower when the mother had an allergy-related disease compared with when the mother did not have an allergy-related disease (yes: 1.73, 95% CI 1.11–2.69 vs no: 1.34, 95% CI 0.81–2.21), and that this phenomenon was particularly pronounced in the group of AD parents (yes: 1.67, 95% CI 0.81–2.21). (yes: 1.67, 95% CI 1.16–2.4 vs no: 0.74, 95% CI 0.25–2.18); in contrast, father’s allergy-related disease significantly increased the risk of breastfeeding for AD (yes: 2.0, 95% CI 1.08–3.67 vs no: 1.26, 95% CI 0.85–1.87). The risk effect of 3 or more antibiotic administrations on AD was significantly lower when the father had an allergy-related disease compared to when the father did not have an allergy-related disease (yes: 1.66, 95% CI 0.77–3.58 vs no: 2.3, 95% CI 1.42–3.73). This phenomenon was particularly significant in the group of parents with AD (yes: 0.91, 95% CI 0.11–7.35 vs no: 1.8, 95% CI 1.16–2.8). Consistent results regarding the protective effect of older siblings and low birth weight on childhood AD were demonstrated in the stratification of parental history of allergy-related disease, that is, a parental history of allergy-related disease would significantly reduce this protective effect compared to no history of related disease. Interestingly, the protective effect of low birth weight on AD was significantly reduced in the subgroup with no parental history of AD (OR = 1.13, 95% CI 0.48–2.65) compared with a parental history of AD (OR = 0.62, 95% CI 0.45–0.86), even with the direction of the β coefficient reversed.

### Machine learning model building and feature evaluation

**Fig. 4:**
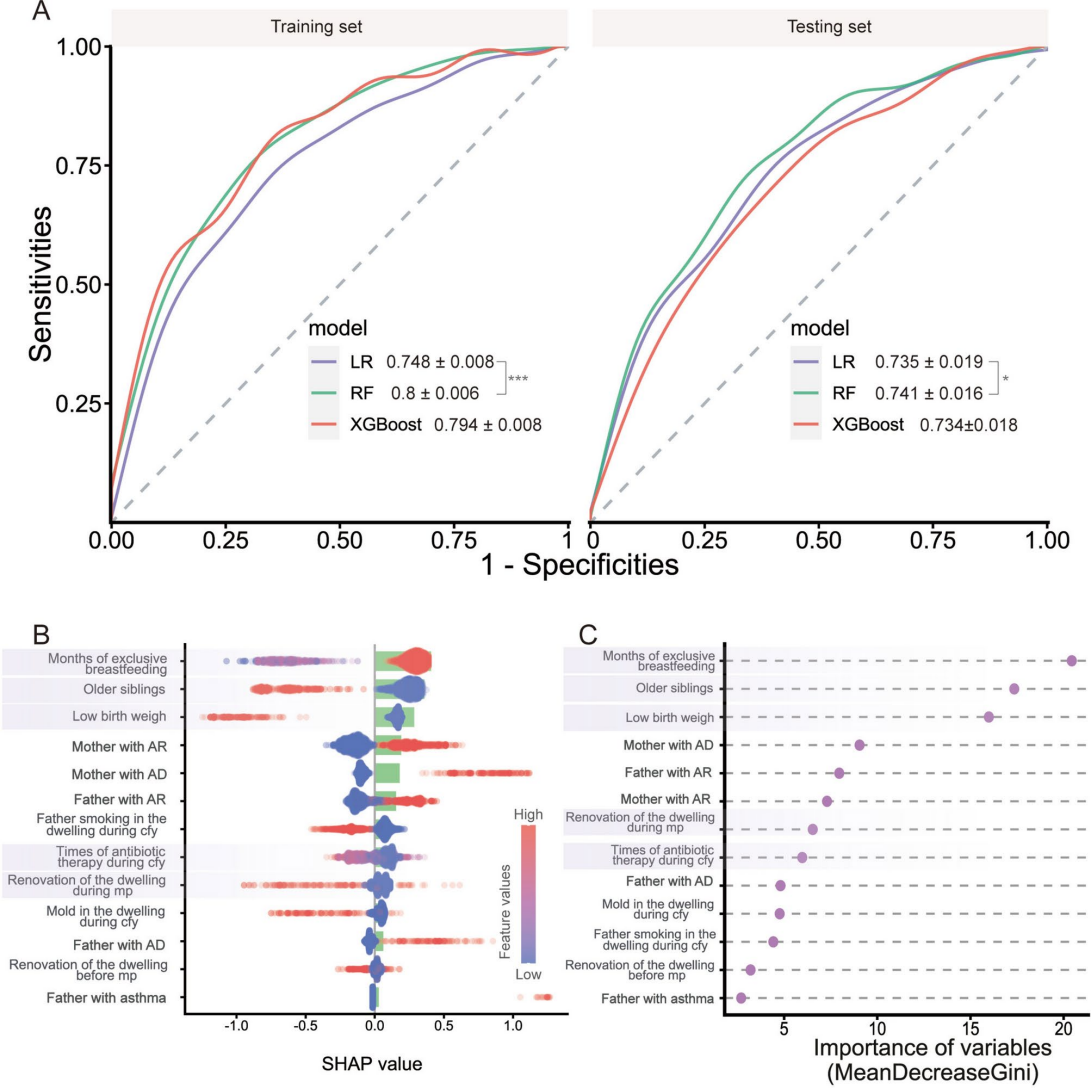
Interpretable machine learning applied to downsampling 1:1 matched case–control data, utilizing logistic regression (LR), random forest (RF), and extreme gradient boosting (XGBoost) models. The five key variables indicated in Fig. 2 include new renovations during maternal pregnancy (mp), frequency of antibiotic therapy during the first year (cfy), duration of exclusive breastfeeding, presence of older siblings, and low birth weight were highlight in the plot. (**A**) Area under the receiver operating characteristic (AUROC) curves for the training and testing sets of the three models across 100 iterations. (**B**) SHAP value evaluation based on the testing dataset from the XGBoost model. Each point represents a sample, with point color indicating the value of the corresponding feature. The dichotomous variable (e.g., older siblings) is represented by distinct colors, while the multicategorical ordinal variable (e.g., months of exclusive breastfeeding) reflects gradient color based on category count; affiliated feature SHAP values are indicated by green bars. SHAP values greater than zero indicate a positive contribution to the outcome, reflecting a hazard effect. For example, children with older siblings (coded as 1) are less likely to develop AD than those without (coded as 0). (**C**) Mean Decrease Gini (MDG) size assessment from the full random forest (RF) model; larger MDG values indicate greater feature importance. *mp* maternal pregnancy, *cfy* child’s first year, *AR* allergic rhinitis, *AD* atopic dermatitis.

We obtain the optimal parameters of the models through a grid search strategy, and details about the hyperparameters of the relevant models and their explanations can be found in Table S5. Figure 4A shows the average performance of the three models over 100 iterations on both the training set and the testing set. It can be seen that the Random Forest model performs optimally and significantly better than the Logistics regression model in both the training set (AUROC: 0.80 ± 0.006) and the testing set (AUROC: 0.741 ± 0.016); the specific performances of the three models and their hypothesis tests are shown in Table S6. Figure 4B,C show that under the optimal parameters, the global importance assessment of XGBoost and Random Forest models on downsampled data. Both SHAP and MDG values suggest that breastfeeding duration, older siblings, and low birth weight occupy considerable importance. Also, Fig. 4B suggests that exclusive breastfeeding for 4 months and above (Code: 3) increases AD risk; whereas having older siblings (Code: 1) and low birth weight (Code: 1) reduces children’s AD risk. Finally, we also built an online AD prediction tool (https://admodel-ghrcirrmt5ik6wz5shqydj.streamlit.app/) relying on the RF model of Fig. 4C to identify early the risk of AD in children aged 2–8 years using early life variables.

## Discussion

About 20% of children and 10% of adults worldwide suffer from AD, a multifactorial inflammatory skin condition that lowers the quality of life and has a non-negligible economic impact^2,8^. AD is prevalent in children and often precedes a cascade of allergic conditions, including food allergies, allergic rhinitis, and asthma^31^. A recent study reported a 12.0% prevalence of AD in preschool-aged children in Urumqi^32^. Apart from causing discomfort and itching, AD can significantly impact children’s self-esteem and future social engagement^3,4^. Therefore, it is crucial to investigate early infancy risk factors for AD to facilitate timely prevention and treatment. This case–control study revealed that exclusive breastfeeding for 4 months or above, antibiotics using during child’s first year for 3 times or above and renovation of the dwelling during mother’s pregnancy have dangerous impact on the development of AD in children aged 2–8 years. Conversely, having older siblings and low birth weight (< 2.5 kg) were found to be protective factors for AD.

One significant risk factor for atopic disease in children is the history of atopic disease in either or both parents. For example, prospective studies have revealed that a high FLG mutation risk score (OR = 1.8; 95% CI 1.1–2.9), parental asthma (OR = 3.7; 95% CI 1.2–11.5), and parental AD (OR = 6.2; 95% CI 1.2–23.2) are substantial genetic risks for persistent AD in children^11^. A significant prospective cohort study conducted at the beginning of the century discovered that children whose parents had atopic disease had an increased risk of AD by the time the children were 4 years old; the risk of AD in children with a parental atopic history was nearly twice as high as that in children without a parental atopic history^33^; this effect was even more pronounced when parental AD was taken into account. The present research discovered that atopic disease of the parents, such as atopic dermatitis, asthma, and allergic rhinitis, all exacerbated the risk of AD in preschoolers, particularly in mothers. This is also evidenced by the assessment of the factors’ importance by predictive models.

Multivariate regression analysis identified that renovation of the dwelling during mother’s pregnancy increase the AD risk in children, compared to the period before the mother’s pregnancy and the child’s first year. The release of chemicals such as formaldehyde, organic volatiles, surfactants, and environmental endocrine disruptors (EDCs) during the renovation process has been linked to adverse health effects, particularly in young children and infants^34^. Additionally, immunological research suggests a higher prevalence of the type 2 helper cell phenotype in AD patients, characterized by elevated serum IgE and interleukin (IL)-4 levels. Furthermore, decorative and furniture materials containing volatile organic compounds (VOCs) can impact the fetal immune system and compromise the skin barrier, intensifying the sensitization process to indoor dust mites and molds^35–37^. This is particularly significant during the seventh to seventeenth month of gestation, a crucial period for fetal epidermal differentiation and susceptibility to exogenous hazardous substances such as PM2.5^37^. Overall, the negative impacts of new renovations during the mother’s pregnancy are significantly associated with the subsequent development of AD in children, compared to both new renovations before and after pregnancy.

The association between antibiotic use and childhood AD has been extensively studied, yielding varying conclusions due to differences in study design, periods of interest, and antibiotic types and doses, which have impaired the credibility of meta-analyses^38,39^. A multicenter cross-sectional study revealed a correlation (OR = 1.20, 95% CI 1.11–1.30) between childhood AD and antibiotic use in children aged 0–1 years^40^. Similarly, a retrospective cohort study found that prenatal antibiotic exposure increased the incidence of AD in 11-year-old children (aHR = 1.19, 95% CI 1.09–1.31)^41^. However, most studies have not shown an adverse impact or a statistically significant link between antibiotic usage in neonates and childhood AD^22,42^. For example, a retrospective study based on a prospective cohort found decreased odds ratios for developing AD in children using antibiotics during the ages of 0–1 year and 1–4 years, with OR of 0.61 and 0.11, respectively; as well as a lower risk of atopic sensitization, with OR of 0.38 and 0.15, respectively^22^. Moreover, the quantity and frequency of antibiotic use have not been thoroughly examined in many studies positively associated with AD^43^. This study found a significant association between antibiotic use in the first year of life and AD in preschool-aged children, demonstrating a frequency-enhanced effect (P for trend < 0.001). Furthermore, multivariable analysis indicated that administering three or more antibiotic doses between 0 and 1 year is associated with a 92% increased risk of AD, regardless of the child’s mode of birth or parental history of atopic disorders. However, stratified analyses revealed that the risk of AD associated with the use of three or more antibiotics was significantly reduced in children of parents with a history of AD, even becoming non-significant compared to those whose parents did not have AD. While the exact mechanism remains unknown, this phenomenon suggests complex interactions between antibiotic use and parental AD that may influence the development of AD in children. These findings provide important implications for further research on antibiotic use.

Preliminary research indicates a possible preventive effect of breastfeeding on childhood atopic dermatitis. A meta-analysis of prospective studies published prior to 2000 found that exclusive breastfeeding for at least three months reduced the risk of AD, especially when accounting for parental atopic disorders (OR = 0.58; 95% CI 0.41–0.92)^44^. However, recent studies have not provided adequate evidence to support the protective role of exclusive breastfeeding against AD^45^. Conversely, a 2014 Japanese birth cohort study reported an increased risk of AD associated with exclusive breastfeeding compared to formula feeding (OR = 1.26, 95% CI 1.12–1.41), showing a dose–response relationship (P for trend < 0.001)^46^. Several factors, such as recall bias, study design, and varying interpretations of exclusive breastfeeding, may account for this discrepancy. Additionally, exclusive breastfeeding may not supply adequate vitamin D, potentially leading to deficiency and an increased risk of AD; however, vitamin D supplementation can improve clinical symptoms of AD^47,48^. Moreover, recent advancements in infant formula may have diminished some benefits of breastfeeding. The hygiene hypothesis posits that prolonged exclusive breastfeeding decreases exposure to pathogenic stimuli, favoring type 2 helper cells over type 1 cells and increasing the risk of allergy development^13^. Furthermore, it proposes that breast milk contains antimicrobial and anti-inflammatory bioactive compounds that enhance infant resistance to infections^49^. Introducing a diverse array of complementary foods between 6 and 12 months helps establish intestinal flora homeostasis, thereby reducing AD incidence from 1 to 2 years of age^50^. Our study identified a 1.59-fold increased risk of AD in children exclusively breastfed for four months or longer compared to their non-breastfed counterparts. Further stratified analyses indicated that this effect was most pronounced in children without a parental history of atopic disease, particularly when the mother lacked such a history. This finding suggests that mothers with a history of allergy-related diseases may possess specific antibodies or molecules that confer some protection against AD, allowing the child to acquire a degree of resistance through breastfeeding. This hypothesis warrants further exploration in relevant basic research. A recent matched case–control study indicated a significantly lower risk of AD in children under two years old when weaned or introduced to a diverse solid complementary diet as early as 4 months, with ORs of 0.41 (95% CI 0.20–0.87) and 0.30 (95% CI 0.11–0.81), respectively^51^. However, this effect diminished after stratification by the child’s mode of birth in our stratification analysis, implying that the mode of birth may be a confounding factor influencing the impact of extended exclusive breastfeeding on AD. This necessitates further rigorous studies for confirmation.

The hygiene hypothesis, proposed in 1989, has undergone significant revisions and modifications. Strachan initially suggested that the exchange of early childhood infections between siblings could protect against immune-related diseases^26^. Building on this concept, Rook introduced the “Old-Friends-Hypothesis,” which emphasizes the coexistence of infectious diseases and human evolution over time, suggesting that appropriate early-life exposure to microbial communities can help prevent immune-related diseases and allergic conditions^52^. Subsequent Alpine farm studies provided strong evidence for this hypothesis, broadening our understanding of the relationship between health and early-life microbial exposure^53^. In the field of immunology, the Microbiota Hypothesis, developed by Noverr and Hufnagle, has been refined through the study of microbial communities and their interactions with host mucosal surfaces, highlighting their metabolic and immunological effects^54^. Simultaneously, phylogenetic evidence indicates a lower variety and richness of microbial communities in invertebrates compared to vertebrates^55^. Combining concepts from biological evolution, immunology, and microbiology, the hygiene hypothesis is considered a historically relevant model explaining how modern lifestyles impact human health. It emphasizes the long-evolved balance between pathogen stimuli and immune responses from a human-nature perspective, suggesting a potential link to the rising prevalence of allergy-related diseases in industrialized nations^56,57^. Notably, this study found that children with older siblings had a 24% lower risk of AD, independent of parental atopic disease. However, no correlation was found between having a dog at home and AD in children aged 0–1 year. According to the hygiene hypothesis, close contact with older siblings, whether in caregiving person, may increase a child’s exposure to pathogenic stimuli after birth, potentially lowering the risk of AD by promoting normal immune system maturation. A recent study on early-life illnesses and the development of AD in children suggests that older siblings may act as “microbe contact carriers” when interacting with the child^58^. By contrast, children under one year old are less likely to often interact with a dog, and the influence of this relationship is also not significant.

This study suggests that children born with low birth weight have a significantly lower risk of developing AD later in life. This unexpected finding aligns with the notion that babies with low birth weights require more personalized attention. Notably, low birth weight children are less likely than those with normal birth weight to experience exclusive breastfeeding (Table S7), indirectly supporting this observation. Further logistic analyses indicated that low birth weight children were 1.46 times more likely than those with normal birth weight to experience discontinuous exclusive breastfeeding (Table S8). According to the hygiene hypothesis, it is presumed that low birth weight babies may have a reduced risk of AD due to less exclusive breastfeeding and increased nursing care. However, it is evident that multiple factors, like socioeconomic factors and the quality of postnatal nursing care, contribute to this effect, necessitating careful consideration of the role and significance of the hygiene hypothesis in this context.

Artificial intelligence (AI) technologies leveraging machine learning are set to revolutionize atopic dermatitis management by enabling data-driven, personalized treatment. Beyond clinical diagnosis and prognosis, machine learning has increasingly been applied to population studies. For instance, a recent large-scale case–control study utilized machine learning models to extract new insights regarding breast cancer risk factors^59^. In this study, paternal asthma emerged as the weakest predictor of AD among all assessed variables, as indicated by both SHAP values and MeanDecreaseGini scores. However, logistic regression revealed that paternal asthma ranked second to maternal AD in terms of odds ratio (OR) for childhood AD (Table S1). This discrepancy arises because traditional OR measure the ratio of exposure odds in cases versus controls, neglecting the exposure’s actual contribution to the outcome. In contrast, machine learning algorithms focus on the degree of each exposure’s contribution relative to all factors. Both machine learning approaches identified the same key variables: months of exclusive breastfeeding, having older siblings, low birth weight, maternal AD or AR, and paternal AR. Overall, machine learning feature evaluation suggests three key considerations: firstly, the effects of older siblings and hygiene hypotheses on atopic dermatitis warrant adequate attention; secondly, children of parents with AD or AR are a key population of concern for future AD preventive health care, and parents with atopic disease should raise health awareness to prevent the occurrence of AD in their children; lastly, high-quality epidemiological and mechanistic studies are essential to elucidate the impacts of exclusive breastfeeding duration and low birth weight on AD, providing a scientific foundation for maternal and child health practices.

Using a large case–control study, we analyzed the effects of early-life indoor environmental factors, frequency of antibiotic use in infants aged 0–1 years, duration of exclusive breastfeeding, and sibling status on AD in preschool children. During data transformation for stratified analysis and machine learning, we utilized a 1:4 matched dataset, converting it to a 1:1 matched dataset to enhance comparability and strengthen the conclusions. Although our strengths lie in comprehensive analysis and machine learning model development, the study has several limitations. Notably, this is a single-center study conducted in Urumqi and may not fully represent national demographics. As a questionnaire-based retrospective study, potential recall and reporting biases may undermine the scientific rigor of the findings. Furthermore, the stratified analysis and machine learning transformation processes, while ensuring internal data consistency, reduced the representativeness of the control group, widened the gap from real-world data, and challenged the model’s generalizability.

## Conclusion

Our case–control study found that a history of parental atopic disease, indoor renovations during pregnancy, exclusive breastfeeding for four months or longer, and the use of antibiotics three or more times in a child’s first year significantly increased the risk of AD in preschoolers. Conversely, having older siblings and low birth weight were negatively associated with the risk of AD, supporting the hygiene hypothesis. Additionally, our machine learning analysis identified children of parents affected by AD or allergic rhinitis as a priority population for future preventive healthcare. Parents with AD or AR must enhance health awareness to mitigate AD risk in their children. Moreover, reducing antibiotic overuse may be crucial for managing childhood AD. High-quality epidemiological and mechanistic studies are essential to further elucidate the impact of exclusive breastfeeding duration on AD and its underlying mechanisms, offering a scientific basis for maternal and child healthcare practices. Importantly, our findings do not diminish the significance of breastfeeding for healthy child development; rather, they suggest that the role of alternative factors, including supplemental formula, warrants further investigation as society evolves and becomes more affluent. Additionally, the duration of exclusive breastfeeding may need to be redefined.

## Supplementary Information

Supplementary Information 1.

Supplementary Information 2.

Supplementary Information 3.

Supplementary Information 4.

## Data Availability

The datasets generated and/or analyzed during the current study are not publicly available because the data belong to School of Nursing and Health Management, Shanghai University of Medicine and Health Sciences. However, they are available from the corresponding author upon reasonable request. The relevant code used in this article has been uploaded to Github (https://github.com/a-silliy-sheep/AD_model/tree/master/Paper_code).

## References

[1] Ständer, S. Atopic dermatitis. *N. Engl. J. Med.* **384**(12), 1136–1143 (2021). 33761208 10.1056/NEJMra2023911

[2] Laughter, M. R. et al. The global burden of atopic dermatitis: Lessons from the Global Burden of Disease Study 1990–2017. *Br. J. Dermatol.* **184**(2), 304–309 (2021). 33006135 10.1111/bjd.19580

[3] Zuberbier, T. et al. Patient perspectives on the management of atopic dermatitis. *J. Allergy Clin. Immunol.* **118**(1), 226–232 (2006). 16815160 10.1016/j.jaci.2006.02.031

[4] Holm, E. A., Esmann, S. & Jemec, G. B. The handicap caused by atopic dermatitis–sick leave and job avoidance. *J. Eur. Acad. Dermatol. Venereol.* **20**(3), 255–259 (2006). 16503882 10.1111/j.1468-3083.2006.01416.x

[5] Drucker, A. M., Wang, A. R. & Qureshi, A. A. Research gaps in quality of life and economic burden of atopic dermatitis: The National Eczema Association burden of disease audit. *JAMA Dermatol.* **152**(8), 873–874 (2016). 27305113 10.1001/jamadermatol.2016.1978

[6] Silverberg, J. I. Health care utilization, patient costs, and access to care in US adults with eczema: A population-based study. *JAMA Dermatol.* **151**(7), 743–752 (2015). 25738422 10.1001/jamadermatol.2014.5432

[7] Lee, C. K., Chang, C. C., Johar, A., Puwira, O. & Roshidah, B. Fingerprint changes and verification failure among patients with hand dermatitis. *JAMA Dermatol.* **149**(3), 295–299 (2013). 23682364 10.1001/jamadermatol.2013.1425

[8] Arents, B. W. M., van Zuuren, E. J., Vermeulen, S., Schoones, J. W. & Fedorowicz, Z. Global Guidelines in Dermatology Mapping Project (GUIDEMAP), a systematic review of atopic dermatitis clinical practice guidelines: Are they clear, unbiased, trustworthy and evidence based (CUTE)?. *Br. J. Dermatol.* **186**(5), 792–802 (2022). 34984668 10.1111/bjd.20972 PMC9325494

[9] van Zuuren, E. J., Fedorowicz, Z., Christensen, R., Lavrijsen, A. & Arents, B. W. M. Emollients and moisturisers for eczema. *Cochrane Database Syst. Rev.* **2**(2), CD012119 (2017). 28166390 10.1002/14651858.CD012119.pub2 PMC6464068

[10] Ramírez-Marín, H. A. & Silverberg, J. I. Differences between pediatric and adult atopic dermatitis. *Pediatr. Dermatol.* **39**(3), 345–353 (2022). 35297082 10.1111/pde.14971

[11] Thorsteinsdottir, S. et al. Genetic, clinical, and environmental factors associated with persistent atopic dermatitis in childhood. *JAMA Dermatol.* **155**(1), 50–57 (2019). 30427975 10.1001/jamadermatol.2018.4061 PMC6439574

[12] Rupnik, H., Rijavec, M. & Korošec, P. Filaggrin loss-of-function mutations are not associated with atopic dermatitis that develops in late childhood or adulthood. *Br. J. Dermatol.* **172**(2), 455–461 (2015). 25314673 10.1111/bjd.13477

[13] Sherriff, A., Golding, J., Alspac Study Team. Hygiene levels in a contemporary population cohort are associated with wheezing and atopic eczema in preschool infants. *Arch. Dis. Child.* **87**(1), 26–29 (2002). 12089117 10.1136/adc.87.1.26 PMC1751124

[14] To, T. et al. Early life exposure to air pollution and incidence of childhood asthma, allergic rhinitis and eczema. *Eur. Respir. J.* **55**(2), 1900913 (2020). 31806712 10.1183/13993003.00913-2019 PMC7031706

[15] Thyssen, J. P., Zirwas, M. J. & Elias, P. M. Potential role of reduced environmental UV exposure as a driver of the current epidemic of atopic dermatitis. *J. Allergy Clin. Immunol.* **136**(5), 1163–1169 (2015). 26298230 10.1016/j.jaci.2015.06.042

[16] Hansel, T. T., Johnston, S. L. & Openshaw, P. J. Microbes and mucosal immune responses in asthma. *Lancet* **381**(9869), 861–873 (2013). 23428115 10.1016/S0140-6736(12)62202-8

[17] Grüber, C., Warner, J., Hill, D., Bauchau, V., EPAAC Study Group. Early atopic disease and early childhood immunization—is there a link?. *Allergy* **63**(11), 1464–1472 (2008). 18925883 10.1111/j.1398-9995.2008.01696.x

[18] Cilluffo, G. et al. Associations of greenness, greyness and air pollution exposure with children’s health: A cross-sectional study in Southern Italy. *Environ. Health* **17**(1), 86 (2018). 30518403 10.1186/s12940-018-0430-x PMC6282291

[19] Asher, M. I. et al. Worldwide time trends in the prevalence of symptoms of asthma, allergic rhinoconjunctivitis, and eczema in childhood: ISAAC Phases One and Three repeat multicountry cross-sectional surveys. *Lancet* **368**(9537), 733–743 (2006). 16935684 10.1016/S0140-6736(06)69283-0

[20] Strachan, D. P., Harkins, L. S. & Golding, J. Sibship size and self-reported inhalant allergy among adult women. ALSPAC Study Team. *Clin. Exp. Allergy* **27**(2), 151–155 (1997). 9061213

[21] Benn, C. S., Melbye, M., Wohlfahrt, J., Björkstén, B. & Aaby, P. Cohort study of sibling effect, infectious diseases, and risk of atopic dermatitis during first 18 months of life. *BMJ* **328**(7450), 1223 (2004). 15121716 10.1136/bmj.38069.512245.FE PMC416593

[22] Dom, S. et al. Pre- and post-natal exposure to antibiotics and the development of eczema, recurrent wheezing and atopic sensitization in children up to the age of 4 years. *Clin. Exp. Allergy* **40**(9), 1378–1387 (2010). 20545699 10.1111/j.1365-2222.2010.03538.x

[23] Gassner-Bachmann, M. & Wüthrich, B. Bauernkinder leiden selten an Heuschnupfen und Asthma [Farmers’ children suffer less from hay fever and asthma]. *Dtsch. Med. Wochenschr.* **125**(31–32), 924–931 (2000). 10967955 10.1055/s-2000-6778

[24] Remes, J. A., Castro-Rodriguez, Holberg, C. J. et al. Pet exposure in infancy and wheeze and asthma in childhood. In *Proceedings of the 96th American Thoracic Society Meeting* (2000).

[25] Bach, J. F. The effect of infections on susceptibility to autoimmune and allergic diseases. *N. Engl. J. Med.* **347**(12), 911–920 (2002). 12239261 10.1056/NEJMra020100

[26] Strachan, D. P. Hay fever, hygiene, and household size. *BMJ* **299**(6710), 1259–1260 (1989). 2513902 10.1136/bmj.299.6710.1259 PMC1838109

[27] Dehdar, S. et al. Applications of different machine learning approaches in prediction of breast cancer diagnosis delay. *Front. Oncol.* **13**, 1103369 (2023). 36874113 10.3389/fonc.2023.1103369 PMC9978377

[28] Ha, R. et al. Convolutional neural network based breast cancer risk stratification using a mammographic dataset. *Acad. Radiol.* **26**(4), 544–549 (2019). 30072292 10.1016/j.acra.2018.06.020 PMC8114104

[29] Van Buuren, S. & Groothuis-Oudshoorn, K. mice: Multivariate imputation by chained equations in R. *J. Stat. Softw.* **45**, 1–67 (2011).

[30] Lundberg, S. M. et al. Explainable machine-learning predictions for the prevention of hypoxaemia during surgery. *Nat. Biomed. Eng.* **2**(10), 749–760 (2018). 31001455 10.1038/s41551-018-0304-0 PMC6467492

[31] Hahn, E. L. & Bacharier, L. B. The atopic march: The pattern of allergic disease development in childhood. *Immunol. Allergy Clin. North Am.* **25**(2), 231–v (2005). 15878453 10.1016/j.iac.2005.02.004

[32] Shi, H. et al. Prevalence and influencing risk factors of eczema among preschool children in Urumqi city: A cross-sectional survey. *BMC Pediatr.* **21**(1), 347 (2021). 34399722 10.1186/s12887-021-02819-5 PMC8365957

[33] Böhme, M., Wickman, M., Lennart Nordvall, S., Svartengren, M. & Wahlgren, C. F. Family history and risk of atopic dermatitis in children up to 4 years. *Clin. Exp. Allergy* **33**(9), 1226–1231 (2003). 12956743 10.1046/j.1365-2222.2003.01749.x

[34] Wang, I. J., Lin, C. C., Lin, Y. J., Hsieh, W. S. & Chen, P. C. Early life phthalate exposure and atopic disorders in children: A prospective birth cohort study. *Environ. Int.* **62**, 48–54 (2014). 24161446 10.1016/j.envint.2013.09.002

[35] Lehmann, I. et al. The influence of maternal exposure to volatile organic compounds on the cytokine secretion profile of neonatal T cells. *Environ. Toxicol.* **17**(3), 203–210 (2002). 12112628 10.1002/tox.10055

[36] Huss-Marp, J. et al. Influence of short-term exposure to airborne Der p 1 and volatile organic compounds on skin barrier function and dermal blood flow in patients with atopic eczema and healthy individuals. *Clin. Exp. Allergy* **36**(3), 338–345 (2006). 16499645 10.1111/j.1365-2222.2006.02448.x

[37] Yao, T. C. et al. Association of prenatal exposure to fine particulate matter pollution with childhood eczema. *Allergy* **76**(7), 2241–2245 (2021). 33432626 10.1111/all.14738 PMC8249326

[38] Jordan, S., Storey, M. & Morgan, G. Antibiotics and allergic disorders in childhood. *Open Nurs. J.* **2**, 48–57 (2008). 19319220 10.2174/1874434600802010048 PMC2582823

[39] Ahmadizar, F. et al. Early-life antibiotic exposure increases the risk of developing allergic symptoms later in life: A meta-analysis. *Allergy* **73**(5), 971–986 (2018). 29105784 10.1111/all.13332

[40] Norbäck, D. et al. Sources of indoor particulate matter (PM) and outdoor air pollution in China in relation to asthma, wheeze, rhinitis and eczema among pre-school children: Synergistic effects between antibiotics use and PM10 and second hand smoke. *Environ. Int.* **125**, 252–260 (2019). 30731375 10.1016/j.envint.2019.01.036

[41] McKeever, T. M., Lewis, S. A., Smith, C. & Hubbard, R. The importance of prenatal exposures on the development of allergic disease: A birth cohort study using the West Midlands General Practice Database. *Am. J. Respir. Crit. Care Med.* **166**(6), 827–832 (2002). 12231492 10.1164/rccm.200202-158OC

[42] Kummeling, I. et al. Early life exposure to antibiotics and the subsequent development of eczema, wheeze, and allergic sensitization in the first 2 years of life: The KOALA Birth Cohort Study. *Pediatrics* **119**(1), e225–e231 (2007). 17200248 10.1542/peds.2006-0896

[43] Slob, E. M. A. et al. Early-life antibiotic use and risk of asthma and eczema: Results of a discordant twin study. *Eur. Respir. J.* **55**(4), 1902021 (2020). 32139457 10.1183/13993003.02021-2019

[44] Gdalevich, M., Mimouni, D., David, M. & Mimouni, M. Breast-feeding and the onset of atopic dermatitis in childhood: A systematic review and meta-analysis of prospective studies. *J. Am. Acad. Dermatol.* **45**(4), 520–527 (2001). 11568741 10.1067/mjd.2001.114741

[45] Matheson, M. C., Allen, K. J. & Tang, M. L. Understanding the evidence for and against the role of breastfeeding in allergy prevention. *Clin. Exp. Allergy* **42**(6), 827–851 (2012). 22276526 10.1111/j.1365-2222.2011.03925.x

[46] Ito, J. & Fujiwara, T. Breastfeeding and risk of atopic dermatitis up to the age 42 months: A birth cohort study in Japan. *Ann. Epidemiol.* **24**(4), 267–272 (2014). 24342028 10.1016/j.annepidem.2013.11.007

[47] Furman, L. Maternal vitamin D supplementation for breastfeeding infants: Will it work?. *Pediatrics* **136**(4), 763–764 (2015). 26416926 10.1542/peds.2015-2312

[48] Hattangdi-Haridas, S. R. et al. Vitamin D deficiency and effects of vitamin D supplementation on disease severity in patients with atopic dermatitis: A systematic review and meta-analysis in adults and children. *Nutrients* **11**(8), 1854 (2019). 31405041 10.3390/nu11081854 PMC6722944

[49] Chirico, G., Marzollo, R., Cortinovis, S., Fonte, C. & Gasparoni, A. Antiinfective properties of human milk. *J. Nutr.* **138**(9), 1801S-1806S (2008). 18716190 10.1093/jn/138.9.1801S

[50] Zhong, C. et al. Increased food diversity in the first year of life is inversely associated with allergic outcomes in the second year. *Pediatr. Allergy Immunol.* **33**(1), e13707 (2022). 34843132 10.1111/pai.13707

[51] Turati, F. et al. Early weaning is beneficial to prevent atopic dermatitis occurrence in young children. *Allergy* **71**(6), 878–888 (2016). 26893011 10.1111/all.12864

[52] Rook, G. A. & Brunet, L. R. Microbes, immunoregulation, and the gut. *Gut* **54**(3), 317–320 (2005). 15710972 10.1136/gut.2004.053785 PMC1774411

[53] Riedler, J. et al. Exposure to farming in early life and development of asthma and allergy: A cross-sectional survey. *Lancet* **358**(9288), 1129–1133 (2001). 11597666 10.1016/S0140-6736(01)06252-3

[54] Noverr, M. C. & Huffnagle, G. B. The ‘microflora hypothesis’ of allergic diseases. *Clin. Exp. Allergy* **35**(12), 1511–1520 (2005). 16393316 10.1111/j.1365-2222.2005.02379.x

[55] McFall-Ngai, M. Adaptive immunity: Care for the community. *Nature* **445**(7124), 153 (2007). 17215830 10.1038/445153a

[56] Pfefferle, P. I., Keber, C. U., Cohen, R. M. & Garn, H. The hygiene hypothesis—Learning from but not living in the past. *Front. Immunol.* **12**, 635935 (2021). 33796103 10.3389/fimmu.2021.635935 PMC8007786

[57] Watts, G. The defence of dirt. *BMJ* **328**(7450), 1226 (2004). 15121715 10.1136/bmj.38075.565822.55 PMC416594

[58] Chatenoud, L. et al. Markers of microbial exposure lower the incidence of atopic dermatitis. *Allergy* **75**(1), 104–115 (2020). 31321780 10.1111/all.13990

[59] Dianati-Nasab, M. et al. Machine learning algorithms to uncover risk factors of breast cancer: Insights from a large case-control study. *Front. Oncol.* **13**, 1276232 (2024). 38425674 10.3389/fonc.2023.1276232 PMC10903343

