# Supplementary figures and images for: Exploring the role of breastfeeding, antibiotics, and indoor environments in preschool children atopic dermatitis through machine learning and hygiene hypothesis
Source: Sci Rep. 2025 Mar 21;15:9796. doi: 10.1038/s41598-025-94255-z (PMC11928657; doi:10.1038/s41598-025-94255-z)

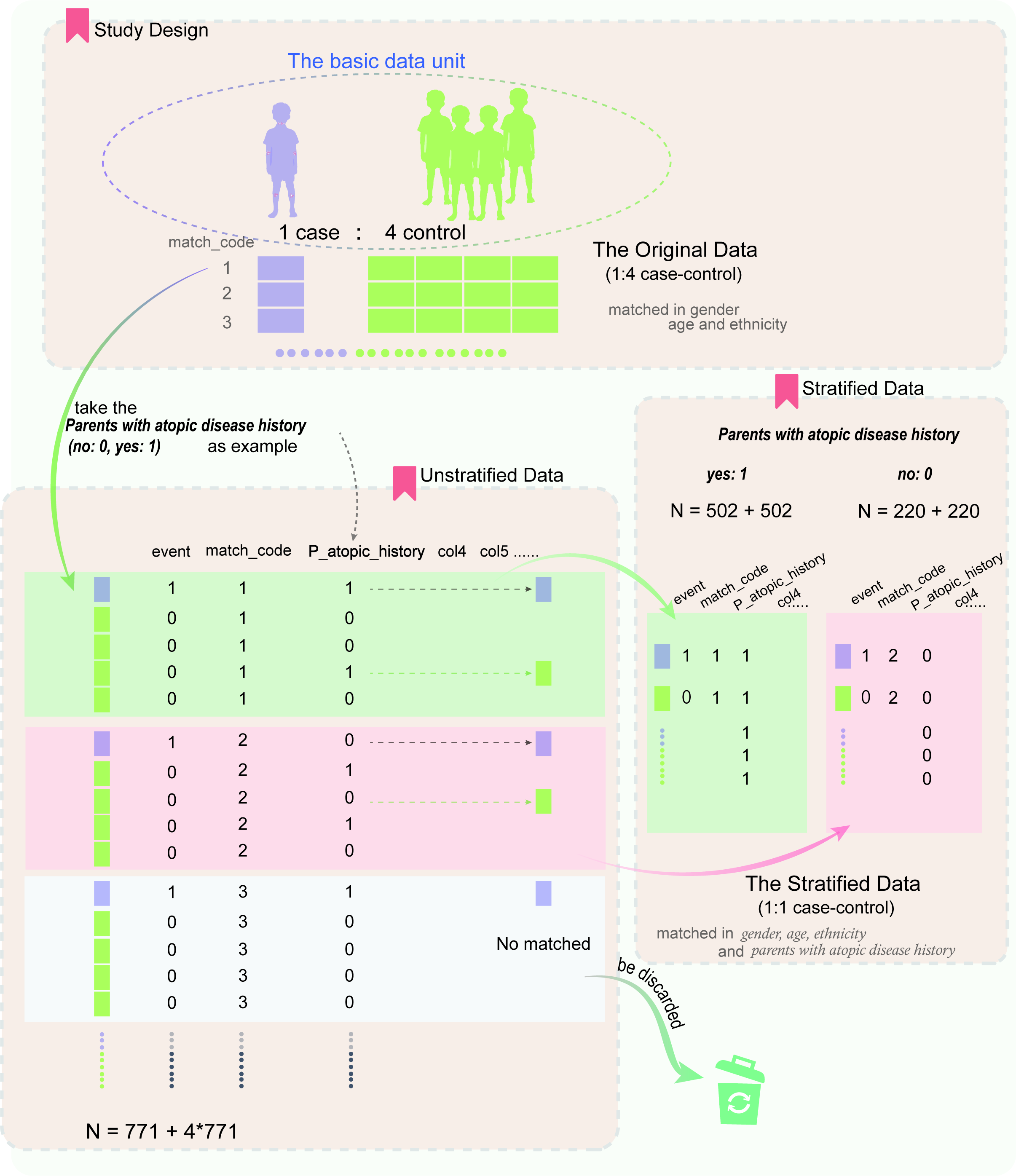

Supplement: Supplementary file 1 — Supplementary Information 1. [file 41598_2025_94255_MOESM1_ESM.tif]

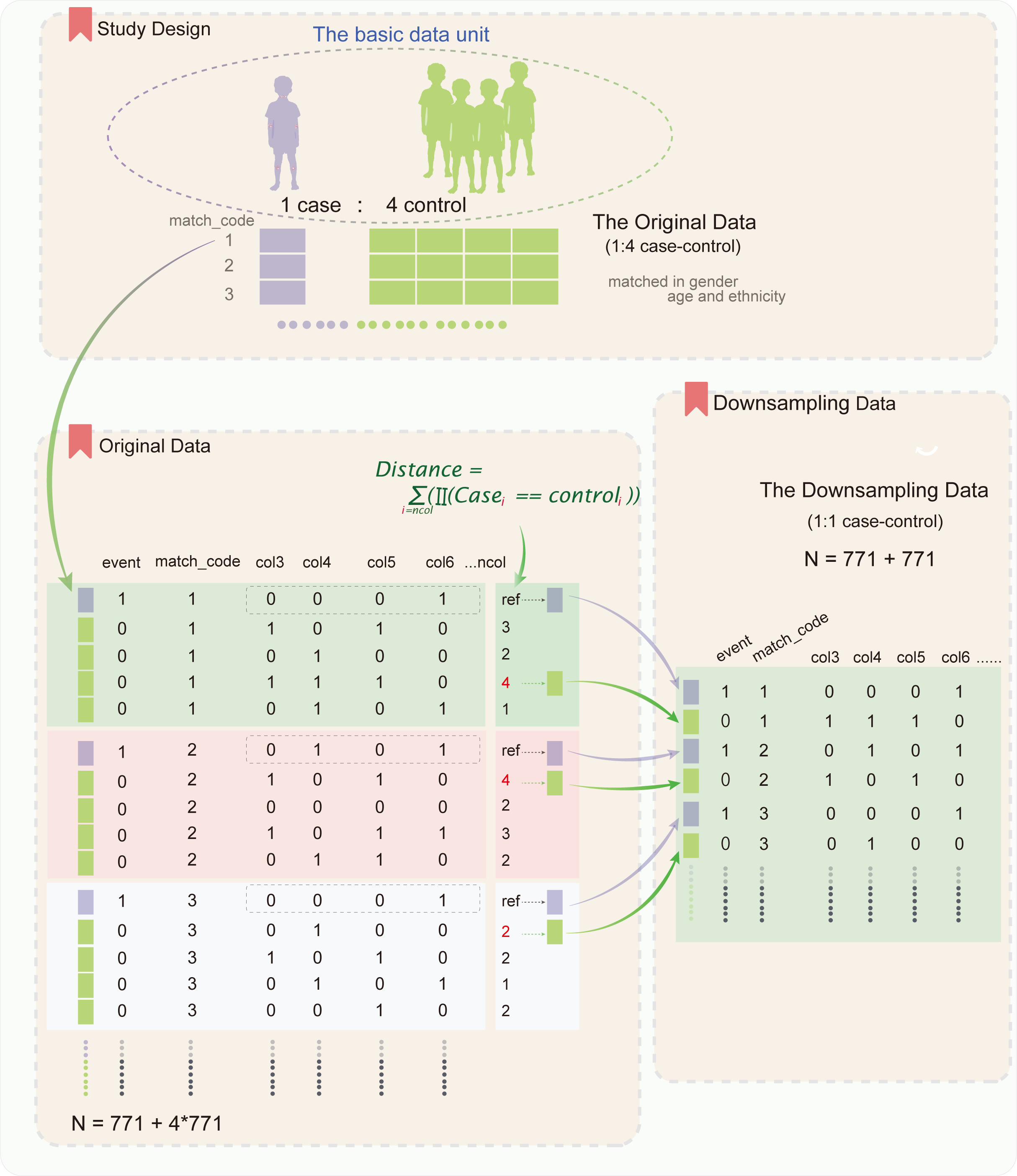

Supplement: Supplementary file 2 — Supplementary Information 2. [file 41598_2025_94255_MOESM2_ESM.tif]
