# Supplementary material for: Exploring the role of breastfeeding, antibiotics, and indoor environments in preschool children atopic dermatitis through machine learning and hygiene hypothesis
Source: Sci Rep. 2025 Mar 21;15:9796. doi: 10.1038/s41598-025-94255-z (PMC11928657; doi:10.1038/s41598-025-94255-z)
Supplement: Supplementary file 4 — Supplementary Information 4. [file 41598_2025_94255_MOESM4_ESM.docx]

**Supplement Table and Figures**

**Table S1.** Logistics analysis of the association between indoor environmental factors in early life, antibiotic use at 0-1 years of age, duration of exclusive breastfeeding, and siblings and AD.

**Table S2.** Stratified analysis of atopic disease history to explore the effect interest factors (including exclusive breastfeeding duration, antibiotic use at age 0-1 years, sibling status, and birth weight) on child AD, corresponding to Figure 3A.

**Table S3.** Stratified analysis of rhinitis or asthma disease history to explore the effect interest factors (including exclusive breastfeeding duration, antibiotic use at age 0-1 years, sibling status, and birth weight) on child AD, corresponding to Figure 3B.

**Table S4.** Stratified analysis of parents with AD and mode of birth to explore the effect interest factors (including exclusive breastfeeding duration, antibiotic use at age 0-1 years, sibling status, and birth weight) on child AD, corresponding to Figure 3C.

**Table S5.** The hyperparameters explanation and values of machine learning model used in this study.

**Table S6.** The hypothesis test of AUROC values between models in training or testing set.

**Table S7**. Univariate analysis of low birth weight and the presence or absence of exclusive breastfeeding.

**Table S8**. Logistic regression of low birth weight and non-exclusive breastfeeding.

**Figure S1.** Illustrations to clarify the stratified analysis strategy. For parents with a history of atopic disease (P_atopic_history), having a parent with AD, allergic rhinitis, or asthma was recorded as 'yes' (coded as 1), while those without were recorded as 'no' (coded as 0). In the first BDU, the P_atopic_history (yes) case sample matched to the third control sample (yes), forming a pair for the 'yes' subgroup of P_atopic_history. Similarly, the P_atopic_history (no) case sample in the second BDU matched the second control sample (no), pairing for the 'no' subgroup. In contrast, the second BDU could not complete matching and was therefore discarded. This process was followed to complete matching across all BDUs.

**Figure S2.** Illustrations clarifying the downsampling strategy for the machine learning. This computational approach transforms 1:4 matched original data into 1:1 matched data, effectively addressing class imbalance issues in model performance.
